# Development, implementation, and evaluation of entrustable professional activities (EPAs) for medical radiation technologists in Taiwan: a nationwide experience

**DOI:** 10.1186/s12909-024-05088-9

**Published:** 2024-01-29

**Authors:** Chun-Yuan Tu, Kuo-Ming Huang, Ching-Hsueh Cheng, Wei-Jou Lin, Cheng-Heng Liu, Chih-Wei Yang

**Affiliations:** 1 Taiwan Association of Medical Radiation Technologists, Taipei, Taiwan; 2 Department of Medical Imaging and Radiological Sciences, I-Shou University, Kaohsiung, Taiwan; 3 Division of Radiation Oncology, Department of Oncology, National Taiwan University Hospital, Taipei, Taiwan; 4 Department of Medical Education, National Taiwan University Hospital, Taipei, Taiwan; 5 Department of Emergency Medicine, National Taiwan University Hospital, Taipei, Taiwan; 6 Department and Graduate Institute of Medical Education and Bioethics, College of Medicine, National Taiwan University, No. 1, Sec. 1, Ren’ai Rd., Zhongzheng Dist., Taipei, 100 Taiwan

**Keywords:** Medical education, Medical radiation technologists, Entrustable professional activities, Competency-based medical education, Faculty development

## Abstract

**Background:**

Competency-based medical education (CBME) is an outcomes-oriented approach focused on developing competencies that translate into clinical practice. Entrustable professional activities (EPAs) bridge competency assessment and clinical performance by delineating essential day-to-day activities that can be entrusted to trainees. EPAs have been widely adopted internationally, but not yet implemented for medical radiation professionals in Taiwan.

**Materials and methods:**

A nationwide consensus process engaged 97 experts in radiation technology education representing diagnostic radiography, radiation therapy, and nuclear medicine. Preliminary EPAs were developed through the focus group discussion and the modified Delphi method. The validity of these EPAs was evaluated using the QUEPA and EQual tools.

**Results:**

Through iterative consensus building, six core EPAs with 18 component observable practice activities (OPAs) in total were developed, encompassing routines specific to each radiation technology specialty. QUEPA and EQual questionnaire data verified these EPAs were valid, and of high quality for clinical teaching and evaluation.

**Conclusion:**

The consensus development of tailored EPAs enables rigorous competency assessment during medical radiation technology education in Taiwan. Further expansion of EPAs and training of clinical staff could potentially enhance care quality by producing competent professionals.

## Introduction

Competency-based medical education (CBME) is an outcome-oriented instructional approach focused on developing and measuring specific competencies that translate into standard professional practices in daily clinical routines [1, 2]. The cornerstone of CBME is competency-based assessment and improvement of trainees’ performance through reliable curricular implementation [3]. CBME has gained widespread advocacy in recent years after emerging in North America in the early 2000s. The paradigm has shifted from time-based to learner-centered, outcome-driven framework [4]. Numerous educational oversight bodies have instituted competency frameworks, including the Accreditation Council for Graduate Medical Education’s (ACGME) Core Competencies in 2002 [5] and the CanMEDS Competency Framework by the Royal College of Physicians and Surgeons of Canada (RCPSC) in 2005 [6]. The ACGME delineated six general competencies: patient care, medical knowledge, interpersonal communication, professionalism, practice-based learning and improvement, and systems-based practice [7]. CBME enables postgraduate clinicians to develop multifaceted abilities, including applied knowledge, attitudes, and skills for clinical reasoning and interprofessional collaboration. Thus, integration of CBME into current medical curricula is imperative.

The concept of entrustable professional activities (EPAs) was first proposed by Ten Cate in 2005 as an implementation strategy for competency-based medical education (CBME) to combine patient care and trainee development [8]. EPAs are defined as “units of professional practice consisting of responsibilities and tasks that supervisors can entrust to trainees once they have demonstrated satisfactory competence“ [9]. In practice, EPAs often comprise a series of tasks to focus the evaluation and performance management process [10], serving as a bridge between clinical practice and competency assessment. EPAs have since been widely adopted in medical education as they delineate essential day-to-day clinical practices. To ensure adequate training outcomes, EPA-based assessments evaluate trainees’ specific knowledge, skills, and attitudes needed for entrustment with for core activities [11].

Adoption of EPAs allows educators to detect trainees’ deficiencies and needs in medical education, leading to their widespread integration in globally [12]. EPAs have also been implemented across numerous residency and physician training programs, including family medicine [13], orthopedic surgery [10], internal medicine [14], emergency medicine [15], anesthesiology [16], pediatrics [17], and psychiatry [18]. More recently, EPA-based education has gained traction among other medical professionals such as nurses [12], pharmacists [19], and physical therapists [20]. In Taiwan, undergraduate students acquire knowledge and skills in medical imaging and radiological science through college education. However, postgraduate curricula are dependent on specific health care settings, including diagnostic radiography, radiation oncology, and nuclear medicine. Based on recent literature searches, there are no reports of EPA implementation in postgraduate training programs for diagnostic radiographers and radiation therapists. Therefore, our study objective is to establish EPAs tailored for radiology departments by achieving consensus among clinical educators. These EPAs are to be designed for integration and supervision within training curricula. By clarifying the principles and key elements of EPAs and providing the proper tools, our project enables clinical teachers to flexibly assess trainees for readiness to act unsupervised, with the ultimate goal of enhancing health services through competent staff trained using the EPA approach.

## Materials and methods

We conducted a nationwide consensus process to develop Entrustable Professional Activities (EPAs) for radiological professionals in Taiwan. The Taiwan Association of Medical Radiation Technologist (TAMRT) collaborated with the National Taiwan University Hospital (NTUH) to engage experts from radiology departments across the country. A total of 97 educators were invited, representing diagnostic radiographers, radiation therapists, and nuclear medicine technologists from various clinical institutions. These experts had extensive teaching and curriculum design experience in postgraduate training programs, and were involved in developing the preliminary EPAs through a focus group discussion and a modified Delphi technique consensus procedures. The preliminary EPAs were then evaluated through surveys and finalized via further expert consensus meetings to establish agreement on the EPAs developed, as well as their use in clinical teaching and assessment. Ultimately, the goal was to improve programmatic assessment and care quality for trainees in medical radiation in Taiwan.

### Supervision levels and OPAs as components of EPAs

EPAs were a concept including observable, measurable and work-based activities and many entrustment scales had been reported in related studies [8]. The crucial question of EPAs is “Do I trust this trainee to accomplish the clinical routine?” Therefore, EPAs should be assessed in number and convert performance of supervision to scale, such as level 1 to 5. The “Level 1” is observation only and “Level 5” is to provide supervision to learners. (Table 1)

**Table 1:** Entrustment levels of EPAs

Trust levels	Contents
Level 1	Observe sideways only
Level 2a	Act in co-activity with a supervisor.
Level 2b	Act alone, but with a supervisor standing by
Level 3a	Indirect supervision, all items checked by a supervisor
Level 3b	Indirect supervision, key items checked by a supervisor
Level 3c	Indirect supervision, key items checked if needed
Level 4	Ability to perform independently
Level 5	Capable of teaching

In our model we distinguished three level of specification for EPAs. The actual EPAs, limited in number, were each specified in three Observable Practice Activities (OPAs), each of which were further described in three to five specifications, to detail what the activities are for which radiation technologist are to be qualified. This adds OPAs between the EPA title and its specification, as recommended in AMEE Guide 140 [21].

Observable Practice Activities (OPAs) [22] were clarified by Warm et al. in 2014 and were described to assess the entrustment with small, specific tasks that the authors called OPAs, at any time to accomplish EPAs development [23]. Given the nature of radiotherapists’ clinical tasks and practice models, workplace observations are typically divided into stages such as before, during, and after the execution of the actual procedure. OPAs were deemed useful to examine these minor elements that enable the workplace observation of different phases within the same EPA. Therefore, OPAs could contribute to the final entrustment decision by aiding in the evaluation process for each comprehensive EPA. In this study, the expert panels were asked to define every EPA and OPA by consensus procedure.

### Consensus-building procedure

A consensus-building procedure utilizing focus group discussion (FGD) and a modified Delphi method was employed to develop topics and content for each EPA. These approaches are generally recommended for consensus-building [24] and aim to achieve agreement and convergence of ideas on a given issue through iterative rounds of inquiry and feedback. FGD is an interactive discussion format that allows all participants the opportunity to express their perspectives for consideration by the group [25]. The following steps were undertaken to develop consensus:

#### Preliminary EPA development

Prior research has demonstrated that developing valid EPAs requires engagement of participants with expertise in the relevant clinical domain and assessment methodology [26]. We recruited a panel of 97 experts across various clinical organizations to design radiological EPAs tailored to key specialty areas, including diagnostic radiographers (DRs), radiation therapists (RTTs), and nuclear medicine technologists (NMTs). The panel experts surveyed relevant literature, including CBME resources [27], existing EPA frameworks in other medical professions [28], and analyzed current training programs. Drawing from this background, they generated a list of critical clinical routines needing entrustment. Following established EPA guidelines [8], the panel formulated titles and descriptions for each proposed EPA. Through this consensus process, a total of 6 preliminary EPAs encompassing 18 observable practice activities (OPAs) were formulated for further discussion and validation.

#### Review and refinement

After developing the initial EPAs, we distributed them to the expert panel members for review to determine if they encompassed the essential clinical skills and adequately covered the necessary attributes. The panel experts were instructed to not only read but closely review the draft EPAs to ensure they contained the requisite attitudes, knowledge, and skills expected of clinical radiological staff. The experts provided feedback by selecting to amend, delete, or retain the proposed EPAs. Additionally, the panelists could suggest modifications or additions to the EPAs or OPAs. All feedback contents were compiled and discussed at the subsequent expert panel meeting. This review process enabled refinement of the EPAs based on the insightful critiques and recommendations of the knowledgeable panel.

#### Final EPA consensus meeting

The focus group discussion (FGD) and modified Delphi method were utilized to conduct the consensus process under the oversight of the Joint Commission of Taiwan (JCT) and TAMRT. A final expert panel meeting was convened in October 2020, including invited DRs, RTTs, and NMTs educators to participate in the EPA consensus building. Prior to the meeting, the preliminary EPAs were distributed to the experts to review the contents and provide input.

During the FGD process, the moderator (C-W Y), a physician educator experienced in consensus methodology and CBME, explained the principles and execution of FGD to achieve a shared mental model among the participants and then applied a standard consensus process for every proposal to moderate the consensus meeting. The moderator reviewed each EPA component generated through FGD and asked for any suggestions for change (i.e., adding, removing, and amending proposals). The proposal for change was seconded by an additional expert before being further discussed, voted on, and documented. Modifications to the drafts were incorporated based on majority expert votes via instant response system (IRS) over 80% to maintain high consensus.

After the FGD process, modified Delphi method was applied to explore the relevance of each EPA, OPA, and their specification. The proposed EPAs, OPAs and their specifications by FGD process were further debated and graded in real-time IRS vote to examine the overall level of agreement on relevance of each item. During the confirmatory process, experts reviewed the description of each EPA, OPA and specification and rated each with respect to the relevance to the training of radiotherapists. In order to gain a high level of concordance among the experts, each item had to meet a quartile deviation of ≤ 0.6 and an average score of ≥ 4 to be included in the final decision. If the criteria for concordance were not met, the items would be left for further debate and review in the next Delphi process round.

#### EPA quality assessment

There are assessment tools to evaluate the quality of EPAs, including the Quality of Entrustable Professional Activities tool (QUEPA) [29] and the EPA Quality tool EQual [30]. We utilized these two methods with 5-point Likert scales to assess the quality of the six developed EPAs specific to each radiology discipline in a faculty development activity. QUEPA and EQual were employed to gauge the effectiveness of the EPAs in meeting evaluable aspects and benchmarks. Additionally, the EQual questionnaire provided insight into the attendants’ level of understanding. Prior research has established an average cutoff score of 4.07 for EPA quality [31, 32]. Thus, any EPA domain in the EQual questionnaire with a mean score below 4.07 would be considered insufficient and likely necessitate revision. This systematic EPA quality assessment ensured the EPAs were robust and meaningful for evaluating clinical competencies.

### Data analysis

The data from the FGD and Delphi consensus processes were collected and analyzed. Questionnaires of QUEPA and EQual were administered to the participants during the faculty development activities. The participants rated their level of agreement on a 5-point Likert scale (1 = strongly disagree, 2 = disagree, 3 = neutral, 4 = agree, and 5 = strongly agree). For each survey, we calculated the mean score and standard deviation (M ± SD) for the ratings.

## Results

The consensus meeting was conducted in October 2020 by convening a national medical education expert panel comprising 97 members (Table 2). The panel included representation from the Departments of Medical Imaging, Radiation Oncology, and Nuclear Medicine. During the FGD process, a total of 48 proposals for change, including 4 adding ones, 39 amending ones and 5 removing ones, were further discussed, voted on to reach consensus, and documented. During the first round Delphi process, all items reached the criteria for concordance and included in the final decisions. Each department planned and consensus on EPAs tailored to their distinct professional domain and finally 6 EPAs, 18 OPAs, and 85 specifications in total were decided. The 6 results EPAs were demonstrated in the Table 3.

**Table 2:** Participant demographics of the consensus meeting

		Expert panel
Attendance		97
Gender		
	Male	51
	Female	46
Institution		
	Medical center	57
	District hospital	36
	Local hospital	4
Working unit		
	Medical imaging	33
	Radiation oncology	31
	Nuclear medicine	33

**Table 3:** Results EPAs, OPAs and their specifications

Diagnostic Radiography		
EPA	OPA	Specifications
EPA1 General Diagnostic Imaging	OPA1 Pre-examination Preparation	1. Confirmation of medical orders and patient identification 2. Explanation of the Inspection Process and Confirmation of Patient Safety 3. Infection Control 4. Radiation Safety Protection
	OPA2 Conducting Imaging Examinations	1. Positioning and Parameter Setting for the Examination 2. Execution of Examinations as Prescribed by Medical Orders 3. Care and Management of Patient Safety 4. Disposition of Adverse Events
	OPA3 Post-Examination Procedures	1. Post-Examination Care Instructions for Patients 2. Environmental Cleaning and Maintenance 3. Reset and Maintenance of Examination Equipment 4. Image Quality Control and Post-Processing
EPA2 Computed Tomography Imaging	OPA1 Pre-examination Preparation	1. Execution of Quality Assurance Operations 2. Confirmation of Medical Orders and Patient Identification 3. Explanation of the Examination Process and Confirmation of Patient Safety 4. Infection Control 5. Radiation Safety Protection
	OPA2 Conducting Imaging Examinations	1. Inspection of Positioning and Parameter Settings 2. Execution of Examinations According to Medical Orders 3. Patient Safety Care and Management 4. Handling of Adverse Events
	OPA3 Post-Examination Procedures	1. Explanation of Post-Examination Care Instructions for Patients 2. Environmental Cleaning and Maintenance 3. Reset and Maintenance of Examination Equipment 4. Image Quality Control and Post-Processing
**Radiation Therapy**		
**EPA**	**OPA**	**Specifications**
EPA1 Computerized Tomography Simulation Positioning	OPA1 Assistive Device Fabrication	1. Confirm medical orders, patient identification, and preparation 2. Provide health education explanations and confirm patient safety 3. Set up and fabricate assistive devices according to medical orders 4. Execute and confirm patient positioning 5. Infection control 6. Patient safety care and management 7. Handling of adverse events
	OPA2 Pre-Positioning Preparation	1. Implement quality assurance procedures 2. Verify medical orders, patient identification, and pre-treatment patient preparation 3. Conduct simple health education briefings and confirm patient safety 4. Infection control 5. Radiation safety and protection
	OPA3 Execution of Simulated Positioning	1. Confirm assistive devices, execute positioning, and set up instrument operations 2. Complete positioning procedures according to medical orders 3. Patient safety care and management 4. Handling of adverse events 5. Provide patients with health education explanations 6. Environmental cleaning and maintenance 7. Handover of patient-related information
EPA2 Remote Radiation Therapy	OPA1 Pre-Treatment Preparation	1. Perform quality assurance operations 2. Confirm medical orders, patient identification, and pre-procedural patient preparation 3. Provide health education instructions and confirm patient safety 4. Infection control 5. Radiation safety and protection
	OPA2 Administer radiation therapy	1. Confirmation of assistive devices, positioning implementation, and instrument operation settings 2. Execution of image-guided verification 3. Completion of treatment procedures as per medical orders 4. Patient safety care and management 5. Handling of exceptional incidents
	OPA3 Post-treatment management	1. Provide patients with health education explanations 2. Environmental cleaning and maintenance 3. Handover of patient-related information
**Nuclear Medicine**		
**EPA**	**OPA**	**Specifications**
EPA1 Positron Emission Tomography (PET) scan	OPA1 Pre-examination Preparation	1. Implementation of quality assurance procedures 2. Verification of medical orders and patient identification 3. Provision of preliminary health education and risk assessment 4. Infection control measures 5. Radiation safety and protection
	OPA2 Conducting Imaging Examinations	1. Instrument positioning and related parameter settings 2. Completion of positron imaging procedures according to medical orders 3. Execution of computed tomography attenuation correction 4. Patient safety care and emergency situation management 5. Assessment and management of other abnormal situations (including team communication)
	OPA3 Post-Examination Procedures	1. Post-examination patient education 2. Environmental cleaning and maintenance 3. Image quality control and abnormal situation management 4. Radiation dose reporting 5. Radiation protection monitoring in the workplace
EPA2 Single Photon Emission Computed Tomography (SPECT) examination	OPA1Pre-examination Preparation	1. Execution of quality assurance operations 2. Confirmation of medical orders and patient identification 3. Preliminary health education briefing and risk assessment 4. Infection control 5. Radiation safety and protection
	OPA2 Conducting Imaging Examinations	1. Setting of instrument-related parameters 2. Completion of single-photon imaging procedures according to medical orders 3. Patient safety care and management of emergencies 4. Assessment and management of other abnormal situations (including team communication)
	OPA3 Post-Examination Procedures	1. Post-examination patient education 2. Environmental cleaning and maintenance 3. Image quality control and management of abnormal situations 4. Radiation protection monitoring in the workplace

Following the consensus meeting, we conducted a faculty development activity. During this event, 192 clinical educators, 119 from the Medical Imaging Department, 35 from the Radiation Oncology Department, and 38 from the Nuclear Medicine Department, evaluated the EPAs employing the EQual and QUEPA questionnaires to determine if the six EPAs developed through expert consensus [33] demonstrated validity. During the session, faculty members from each department assessed the two EPAs within their own respective fields.

The overall QUEPA score data is presented in Table 4. A total of 360 responses were collected, comprised of 222 from the Medical Imaging Department, 67 from the Radiation Oncology Department, and 71 from the Nuclear Medicine Department. This provided quantitative evidence that the established EPAs had validity for training medical radiation professionals.

**Table 4:** QUEPA score for EPAs of different sub-specialties

EPAs for each department QUEPA evaluation form	Medical Imaging		Radiation Therapy		Nuclear Medicine Technology	
	General diagnostic imaging (n = 116)	CT imaging (n = 106)	CT simulation (n = 32)	External beam radiotherapy (n = 35)	PET (n = 35)	SPECT (n = 36)
**Agreements on EPAs naming**	(M ± SD)					
1. The naming is sufficiently focused	4.77 ± 0.42	4.78 ± 0.41	4.56 ± 0.62	4.63 ± 0.55	4.81 ± 0.40	4.92 ± 0.37
2. The scope will not be too broad	4.59 ± 0.66	4.69 ± 0.58	4.59 ± 0.61	4.54 ± 0.56	4.57 ± 0.77	4.72 ± 0.37
3. Describe a single activity	4.65 ± 0.56	4.71 ± 0.55	4.59 ± 0.67	4.69 ± 0.53	4.76 ± 0.60	4.72 ± 0.57
**Observability of EPA missions**						
1. Describe observable actions	4.75 ± 0.45	4.85 ± 0.36	4.53 ± 0.62	4.66 ± 0.59	4.73 ± 0.45	4.81 ± 0.40
2. Clinically common tasks	4.82 ± 0.43	4.84 ± 0.39	4.66 ± 0.55	4.71 ± 0.52	4.78 ± 0.42	4.94 ± 0.23
3. Observability	4.83 ± 0.40	4.89 ± 0.32	4.63 ± 0.55	4.71 ± 0.57	4.86 ± 0.35	4.86 ± 0.35
**Feasibility and generalizability of this EPA mission (for the medical radiation category)**						
1. Are actual clinical practice	4.84 ± 0.36	4.89 ± 0.32	4.69 ± 0.54	4.77 ± 0.49	4.86 ± 0.35	4.92 ± 0.28
2. Activities within the scope of the Medical Radiology	4.82 ± 0.43	4.89 ± 0.40	4.75 ± 0.51	4.83 ± 0.51	4.97 ± 0.16	4.94 ± 0.23
3. Activity that is expected to be performed	4.84 ± 0.39	4.83 ± 0.40	4.63 ± 0.61	4.63 ± 0.60	4.86 ± 0.35	4.81 ± 0.40
4. Can be extended to different practice areas	3.54 ± 1.45	3.53 ± 1.51	4.00 ± 1.11	3.91 ± 1.20	4.41 ± 0.76	4.39 ± 0.87
5. Convertible to different occasions	4.53 ± 0.75	4.46 ± 0.95	4.16 ± 0.88	4.00 ± 1.21	4.54 ± 0.69	4.56 ± 0.69
6. Applicable to different subspecialties	2.87 ± 1.56	2.76 ± 1.55	2.59 ± 1.24	2.89 ± 1.41	4.03 ± 1.26	4.14 ± 1.25
**Underlying Competencies for this EPA Mission**						
1. Contains multiple competencies	4.69 ± 0.58	4.72 ± 0.53	4.56 ± 0.56	4.60 ± 0.65	4.84 ± 0.37	4.92 ± 0.28
2. Can reflect a variety of competencies	4.66 ± 0.60	4.69 ± 0.54	4.53 ± 0.57	4.54 ± 0.61	4.78 ± 0.42	4.86 ± 0.35
3. Need to integrate knowledge, skills and attitudes	4.77 ± 0.48	4.78 ± 0.46	4.66 ± 0.60	4.80 ± 0.53	4.86 ± 0.35	4.92 ± 0.28

As for the EQual questionnaire, 364 responses were obtained, comprised of 224 from the Medical Imaging Department, 64 from the Radiation Oncology Department, and 76 from the Nuclear Medicine Department. The data demonstrated that in the Medical Imaging Department, the overall average score was 4.54 ± 0.63 for the general diagnostic imaging EPAs and 4.48 ± 0.65 for the CT imaging EPAs. In the Radiation Oncology Department, the overall average score was 4.43 ± 0.63 for the CT simulation EPAs and 4.56 ± 0.54 for the external beam radiotherapy EPAs. Finally, in the Nuclear Medicine Department, the overall average score was 4.53 ± 0.57 for the positron emission tomography (PET) imaging EPAs and 4.69 ± 0.49 for the single-photon emission computed tomography (SPECT) imaging EPAs. All item average scores exceeded 4.07 (Table 5). This indicates the final EPAs developed through consensus have high quality and can serve as evaluation guidelines for postgraduate clinical radiation technologist training nationally.

**Table 5:** EQual scores for EPAs of different sub-specialties

EPAs for each department	Medical Imaging		Radiation Therapy		Nuclear Medicine Technology	
Contents of EQual evaluation form	General diagnostic imaging (*n* = 105)	CT imaging (*n* = 119)	CT simulation (*n* = 30)	External beam radiotherapy (*n* = 34)	PET (*n* = 38)	SPECT (*n* = 38)
1. This EPA has a clearly defined starting and ending point	4.89 ± 0.47	4.85 ± 0.53	4.87 ± 0.51	4.94 ± 0.34	4.89 ± 0.45	5.00 ± 0.00
2. This EPA can be independently implemented and achieve clear clinical outcomes	4.67 ± 0.53	4.39 ± 0.76	4.40 ± 0.56	4.62 ± 0.60	4.47 ± 0.65	4.68 ± 0.53
3. This EPA is clear and focused	4.62 ± 0.61	4.38 ± 0.89	4.37 ± 0.72	4.53 ± 0.56	4.37 ± 0.63	4.63 ± 0.63
4. This EPA can be observed in the process	4.65 ± 0.48	4.61 ± 0.51	4.50 ± 0.51	4.71 ± 0.52	4.55 ± 0.50	4.79 ± 0.41
5. The results of this EPA are measurable	4.60 ± 0.51	4.55 ± 0.52	4.50 ± 0.51	4.62 ± 0.49	4.50 ± 0.51	4.76 ± 0.43
6. This EPA is clearly distinguishable from other EPAs in the framework	4.25 ± 0.78	4.13 ± 0.80	4.17 ± 0.75	4.38 ± 0.60	4.11 ± 0.69	4.34 ± 0.71
7. This EPA describes a necessary and important professional task	4.67 ± 0.55	4.68 ± 0.50	4.53 ± 0.57	4.71 ± 0.46	4.58 ± 0.55	4.71 ± 0.52
8. Performance of this EPA demonstrates recognized work outputs or outcomes	4.62 ± 0.54	4.51 ± 0.64	4.37 ± 0.67	4.62 ± 0.49	4.71 ± 0.46	4.82 ± 0.39
9. This EPA is limited to certified personnel in clinical practice	4.42 ± 0.83	4.41 ± 0.81	4.37 ± 0.76	4.47 ± 0.66	4.53 ± 0.69	4.55 ± 0.65
10. This EPA assesses a reliable professional task	4.69 ± 0.61	4.75 ± 0.51	4.73 ± 0.45	4.76 ± 0.50	4.74 ± 0.45	4.82 ± 0.39
11. This EPA requires the application of knowledge, skills or attitudes acquired in training	4.48 ± 0.65	4.49 ± 0.57	4.37 ± 0.61	4.41 ± 0.61	4.71 ± 0.46	4.71 ± 0.46
12. This EPA includes the integration and application of multiple capability-oriented	4.54 ± 0.62	4.61 ± 0.55	4.53 ± 0.73	4.62 ± 0.49	4.66 ± 0.48	4.82 ± 0.39
13. The title of this EPA describes a task rather than a trainee’s traits or abilities	4.39 ± 0.67	4.30 ± 0.68	4.23 ± 0.63	4.35 ± 0.49	4.42 ± 0.50	4.53 ± 0.65
14. Describe a task and avoid adjectives or adverbs that refer to proficiency	4.09 ± 1.01	4.00 ± 0.89	4.07 ± 0.83	4.15 ± 0.78	4.21 ± 0.91	4.55 ± 0.76

## Discussion

The paradigm of medical education is shifting towards competency-based medical education (CBME). Unlike traditional models, CBME is learner-centric and integrates diverse assessments [34, 35]. This philosophical shift has informed pre- and post-graduate curricula internationally [36, 37]. In Taiwan, postgraduate programs have progressively adopted competency-driven approaches with clinical observation. The complexity of clinical environments necessitates that trainees across disciplines develop the requisite knowledge, skills, and attitudes to appropriately manage situations through immersive daily practice and assimilation of professional competencies and responsibilities. Concurrently, clinical educators must observe learners and evaluate their performance and progress multidimensionally. Core competency blueprints [38] and milestone mapping [39, 40] are integral to actualizing CBME. However, focused trainee evaluation requires unified criteria and objectives. EPAs offer an objective tool to appraise competence, enabling professions to delineate and select their field’s most critical, representative clinical skills for guided development.

Integrating EPAs into postgraduate medical radiation technology education presents difficulties. Prior studies largely focused on medicine, including general surgery [41], pediatric cardiology, dentistry [42, 43], and seldom on allied health fields like pharmacy [44] or nursing [45]. This study pioneers EPA implementation as a clinical training assessment across medical radiation technology specialties - diagnostic radiography, radiation therapy, and nuclear medicine.

Medical radiation technologists have different working scopes in different medical institutions, which are affected by the clinical department, the scale of the medical institution, and the execution differences between units. Considering the time pressure, the complexity of the training content, and the differences between different specialties, it takes a lot of time and manpower to develop or change the current evaluation method for trainees. Therefore, at the beginning of the project, we invited course leaders from all levels of medical units across the country to attend courses and discussion meetings to let them understand the core idea of CBME and the EPAs evaluation method. Then, we used the FGD and modified Delphi method to conduct several consensus processes [25, 46], including paper-based data discussions and face-to-face meetings. These two methods provide anonymous and non-hierarchical discussion patterns. Finally, we reached a consensus on six core EPAs tasks, which can be used by each medical unit as the scope and teaching content for evaluating new medical radiation technologists.

EPAs focus on routine clinical behaviors or processes that are performed every day, or clinical activities with high risk and easy to make mistakes. When the assessor has doubts about the completeness of the trainee’s task, or is not confident in a certain clinical skill, it can be reflected in the Entrustment-Supervision (ES) level. Through EPAs evaluation, the course planner can also know if the trainee needs to extend the training period or adjust the course to achieve ability evaluation. EPAs provide a more intuitive assessment of routine medical behaviors. ten Cate recommends dividing supervision into five levels [8]: observation (Level 1), direct supervision (Level 2), indirect supervision and on call for direct help at any time (Level 3), no need for supervision (Level 4) and supervise others (Level 5). Considering the complexity and variability of the content of each radiology profession, we applied the Chen-modified ES scale divided Level 2 direct supervision into joint completion (Level 2a) and timely assistance (Level 2b), and Level 3 indirect supervision into need to confirm all items (Level 3a), key confirmation (Level 3b) and no need to confirm items (Level 3c) [47]. In this way, clinical teachers can more accurately give trainees the corresponding trust level.

This study still has many possible applications that can be explored and extended. As mentioned earlier, the scale and scope of work of medical radiation technologists in various medical units in Taiwan are very different. Although we have invited the course directors and course leaders of the key medical units of all levels in the country, including medical centers, regional hospitals and district hospitals, the number is far from the total number of medical institutions in the country. Through several discussion meetings, we agreed on six EPAs tasks, but the evaluation content and views of these EPAs are not necessarily suitable for all levels of medical units. Therefore, each unit needs to report and feedback to the medical radiation technology organization in charge, so that the content can be modified. In addition, the continuing education of clinical medical radiation technologists after graduation may include multiple aspects. Our team has initially discussed six EPAs tasks, but there are still many EPAs that need to be developed and promoted through consensus process, or the existing EPAs projects need to be carefully planned. This is the direction we need to work hard in the future. The Accreditation Council for Graduate Medical Education (ACGME) demands Clinical competency committees (CCC) to review the clinical performance of trainees in a certain period of time. Team consensus is a necessary process for the development of EPAs in order to determine the clinical training performance and condition of trainees [48, 49]. CCCs can determine the training level to the overall performance of the trainees, and provide feedback to the course directors, so that the follow-up adjustments and processing can be carried out and make summative entrustment decisions about EPAs. We will also move towards the CCC model in the future, so that the training of medical radiation technologists can be more personalized and more complete. We are confident in the results of this study, because it has a great change and far-reaching impact on the training mode and opening of medical radiation technologists in Taiwan. There are still many EPAs related research directions that need to be explored, and our team will continue to explore the development of evaluation tools in the field of medical radiation in Taiwan.

## Data Availability

The datasets generated and analyzed during the current study are not publicly available in order to ensure the privacy of participants. However, they are available from the corresponding author upon reasonable request.
